# Sex differences in the association of metabolic syndrome with low back pain among middle-aged Japanese adults: a large-scale cross-sectional study

**DOI:** 10.1186/s13293-019-0249-3

**Published:** 2019-07-05

**Authors:** Takahiko Yoshimoto, Hirotaka Ochiai, Takako Shirasawa, Satsue Nagahama, Akihito Uehara, Shogo Sai, Akatsuki Kokaze

**Affiliations:** 1 Department of Hygiene, Public Health and Preventive Medicine, Showa University School of Medicine, 1-5-8 Hatanodai, Shinagawa-ku, Tokyo, 142-8555 Japan; 2 All Japan Labor Welfare Foundation, 6-16-11 Hatanodai, Shinagawa-ku, Tokyo, 142-0064 Japan

**Keywords:** Metabolic syndrome, Low back pain, Clustering, Sex difference, Health checkup

## Abstract

**Background:**

Although some recent studies have indicated an association between metabolic syndrome (MetS) and musculoskeletal disease, little is known about the association of MetS with low back pain (LBP). The present study aimed to investigate sex differences in the association of MetS and the clustering of MetS components with LBP among middle-aged Japanese individuals.

**Methods:**

Study subjects were 45,192 adults (30,695 men, 14,497 women) aged 40–64 years who underwent annual health checkups conducted from April 2013 to March 2014. MetS was defined according to the criteria of the Examination Committee of Criteria for MetS in Japan as abdominal obesity plus at least two of dyslipidemia, high blood pressure, or high blood glucose. Information on LBP and health-related lifestyles were collected using a self-administered questionnaire. Logistic regression modeling was used to calculate the odds ratio (OR) and 95% confidence interval (CI) for LBP.

**Results:**

After adjusting for age and lifestyle factors, the OR of MetS for LBP was 1.15 (95% CI 0.95–1.40) in men and 2.16 (95% CI 1.32–3.53) in women. Compared to subjects without abdominal obesity, the presence of abdominal obesity significantly increased the OR for LBP among men (abdominal obesity only: OR 1.34, 95% CI 1.02–1.76; abdominal obesity plus one component: OR 1.24, 95% CI 1.01–1.52; abdominal obesity plus two or more components: OR 1.26, 95% CI 1.02–1.55). Among women, adding other components of MetS to abdominal obesity significantly increased ORs for LBP (abdominal obesity only: OR 1.70, 95% CI 0.94–3.08; abdominal obesity plus one component: OR 1.66, 95% CI 1.06–2.60; abdominal obesity plus two or more components: OR 2.30, 95% CI 1.41–3.78).

**Conclusions:**

This large-scale cross-sectional study indicated that MetS was significantly associated with LBP among women only and that a sex-difference existed in the association between the clustering of MetS components and LBP. Clustering of MetS components by sex may need to be considered for the prevention of LBP, although further prospective studies are needed to clarify the causality.

## Background

Metabolic syndrome (MetS) consists of a cluster of cardiovascular risk factors, and many studies have indicated that MetS was associated with early atherosclerosis [1, 2]. Moreover, accumulation of MetS components is well understood to be associated with increased risks of cardiovascular disease (CVD) and mortality [3–5]. Recently, several studies have investigated associations of MetS with musculoskeletal diseases, including knee osteoarthritis [6], osteoporosis [7], and intervertebral disc degeneration [8].

Low back pain (LBP) is a major musculoskeletal health problem. Globally, LBP represents one of the leading cause of disability [9] and carries an enormous socioeconomic burden including treatment costs and loss of work productivity [10, 11]. LBP is therefore an important public health issue.

Among the multifactorial etiology of LBP, atherosclerosis of the lumbar arteries has been suggested to obstruct blood supply, leading to disc degeneration and LBP [12, 13]. Several studies have reported that subjects with LBP showed more frequently missing or narrowed arteries in the lumbar region [14, 15]. In addition, an association of LBP with abnormal lipid levels has been found in previous studies [16–19]. Moreover, given that LBP and MetS share risk factors including age, obesity, and lifestyle habits [20, 21], MetS could be associated with LBP.

Although several studies have investigated associations between LBP and individual components of MetS such as abdominal obesity [22], hypertension [23], impaired blood glucose [24], and lipid disturbance [17], data on the relationship between LBP and MetS itself are very scarce in the population setting. Only one community-based study has described such associations [25], indicating a significant association between LBP and MetS in women only. However, the effects of the clustering of MetS components on LBP were not investigated in that study. Clarifying the association of LBP with the accumulation of MetS components may have implications for interventions and advice for the prevention of LBP. The aim of the present study was to investigate the associations of LBP with MetS and the clustering of MetS components in a Japanese population using large-scale health checkup data. We analyzed these associations by sex according to a previous study [25] that reported a sex difference in the association of MetS with LBP.

## Methods

### Study population

The present study was designed as a cross-sectional study. Subjects in the present study were men and women aged 40–64 years who underwent an annual health checkup conducted by the All Japan Labor Welfare Foundation, a health checkup center in Japan during the period from April 2013 to March 2014. Of the total of 310,577 subjects who underwent the health checkup and answered the self-administered questionnaire, 310,498 subjects agreed to use their own health checkup data for research. Of these subjects, we excluded 189,156 subjects with missing data for any of the components of MetS. We further excluded 76,150 subjects who did not undergo blood collection in a fasted state (≥ 12 h). As a result, data from 45,192 participants (30,695 men, 14,497 women) were analyzed. Written informed consent for the use of personal information in this study was obtained from all participants. This study protocol was approved by the Medical Ethics Committee of Showa University School of Medicine (Approval No. 2407) and the Ethics Committee of the All Japan Labor Welfare Foundation (Approval No. 9-1-0007).

### Measurements

Demographic information (age, sex), medication use, and health-related lifestyles were investigated using a self-administered questionnaire, which was recommended for specific health checkups by the Ministry of Health, Labour and Welfare in Japan [26]. All participants were required to complete the questionnaire at the time of their health checkup. Information on health-related lifestyles included smoking habits (none, former, current), alcohol intake (none, sometimes, everyday), and regular physical activity equal to walking (≥ 60 min/day or < 60 min/day). LBP was self-reported by the question “Do you have LBP under treatment including follow-up?” [27]. Height, weight, and waist circumference of participants were measured by trained staff. Height and weight were measured in increments of 0.1 cm using a stadiometer, and increments of 0.1 kg using a scale, respectively. Waist circumference was measured to the nearest 0.1 cm at the level of the umbilicus in a standing position. Blood pressure was measured in the sitting position using an automated sphygmomanometer (HEM-907; Omron, Kyoto, Japan). Age was divided into strata of 40–49, 50–59, and 60–64 years for statistical analyses [18].

A venous blood sample was collected from each participant and analyzed within 24 h of being drawn at an external laboratory (SRL, Tokyo, Japan). High-density lipoprotein cholesterol (HDL-C) was measured using a direct method (AU5400; Beckman Coulter, Tokyo, Japan), while triglyceride was determined by an enzyme method (AU5400; Beckman Coulter, Tokyo, Japan). Blood glucose level was obtained by the hexokinase method (AU5400; Beckman Coulter, Tokyo, Japan).

### Definition of MetS

MetS was defined according to the criteria of the Examination Committee of Criteria for Metabolic Syndrome in Japan [28]: abdominal obesity (waist circumference ≥ 85 cm in men, ≥ 90 cm in women) plus at least two of the following three components—(1) dyslipidemia (triglyceride ≥ 150 mg/dL and/or HDL-C < 40 mg/dL and/or medication use for dyslipidemia), (2) high blood pressure (systolic blood pressure ≥ 130 mmHg and/or diastolic blood pressure≥ 85 mmHg and/or taking antihypertensive medication), and (3) high blood glucose (fasting plasma glucose ≥ 100 mg/dL and/or taking antidiabetic medication).

### Statistical analysis

Data are presented as median (25th, 75th percentiles) for continuous variables or as number (percentage) for categorical variables. The chi-square test was used to compare proportions of each component of MetS between groups with and without LBP. To evaluate the relationship between MetS and LBP, a logistic regression model was employed to calculate odds ratios (ORs) and 95% confidence intervals (CIs) for LBP. In the model, age, smoking habits, alcohol intake, and physical activity were included to control for potential confounders [29, 30]. The same logistic regression analyses were then performed to evaluate relationships between accumulation of MetS components and LBP. All statistical analyses were performed using JMP version 13.0 (SAS Institute Japan, Tokyo, Japan). A value of *p* < 0.05 was set as statistically significant, and all reported *p* values are two-sided.

## Results

Mean age (standard deviation) of the participants in this study was 50.5 (7.1) years, and 67.9% were men. Table 1 shows characteristics of study participants by sex. The prevalence of LBP under treatment was 2.1% in men, and 1.7% in women. The prevalence of MetS was 17.6% in men, and 3.5% in women.

**Table 1:** Characteristics of study participants by sex (*n* = 45,192)

	Men (*n* = 30,695)	Women (*n* = 14,497)
Age (years), *n* (%)		
40–49	15,038 (49.0)	6964 (48.0)
50–59	11,152 (36.3)	5565 (38.4)
60–64	4505 (14.7)	1968 (13.6)
Height (cm)	170.0 (165.8, 174.0)	157.3 (153.6, 161.3)
Weight (kg)	68.1 (61.5, 75.4)	53.7 (48.5, 60.5)
Waist circumference (cm)	84.0 (78.5, 90.0)	78.0 (72.0, 84.5)
Smoking habits, *n* (%)		
None	11,746 (38.3)	10,995 (75.9)
Former	6653 (21.7)	1097 (7.6)
Current	12,280 (40.0)	2401 (16.6)
Alcohol intake, *n* (%)		
None	7999 (26.1)	7610 (52.5)
Sometimes	10,190 (33.2)	4543 (31.4)
Everyday	12,489 (40.7)	2336 (16.1)
Physical activity (min/day), *n* (%)		
≥ 60	10,456 (34.1)	4372 (30.2)
< 60	20,183 (65.9)	10,091 (69.8)
Metabolic syndrome, *n* (%)		
+	5402 (17.6)	506 (3.5)
−	25,293 (82.4)	13,991 (96.5)
Low back pain, *n* (%)		
+	641 (2.1)	246 (1.7)
−	30,054 (97.9)	14,251 (98.3)
Systolic blood pressure (mmHg)	128 (117, 141)	119 (108, 134)
Diastolic blood pressure (mmHg)	80 (71, 88)	73 (65, 82)
High-density lipoprotein (mg/dl)	55 (47, 66)	68 (58, 78)
Triglycerides (mg/dl)	104 (72, 155)	71 (53, 101)
Blood glucose (mg/dl)	93 (87, 101)	88 (83, 94)

Values are presented as median (25th and 75th percentile), except where indicated as *n* (%)

Comparisons of each component of MetS between participants with and without LBP are shown in Table 2. The proportion of abdominal obesity was significantly higher in participants with LBP than in those without LBP for each sex (*p* < 0.001). No significant associations were observed in other components of MetS, including dyslipidemia, high blood pressure, and high blood glucose between participants with and without LBP.

**Table 2:** Comparison of each component of metabolic syndrome with or without low back pain

		Low back pain (+) (*n* = 887)	Low back pain (−) (*n* = 44,305)	*p* value^a^
Men				
Abdominal obesity	+	335 (52.3)	13,709 (45.6)	< 0.001
	−	306 (47.7)	16,345 (54.4)	
Dyslipidemia	+	210 (32.8)	9580 (31.9)	0.634
	−	431 (67.2)	20,474 (68.1)	
High blood pressure	+	341 (53.2)	16,009 (53.5)	0.972
	−	300 (46.8)	14,045 (46.7)	
High blood glucose	+	99 (15.4)	4200 (14.0)	0.289
	−	542 (84.6)	25,854 (86.0)	
Women				
Abdominal obesity	+	52 (21.1)	1749 (12.3)	< 0.001
	−	194 (78.9)	12,502 (87.7)	
Dyslipidemia	+	37 (15.0)	1970 (13.8)	0.584
	−	209 (85.0)	12,281 (86.2)	
High blood pressure	+	86 (35.0)	5231 (36.7)	0.573
	−	160 (65.0)	9020 (63.3)	
High blood glucose	+	19 (7.7)	788 (5.5)	0.137
	−	227 (92.3)	13,463 (94.5)	

Data are presented as number (%)

^a^Pearson’s chi-squared test

Crude and adjusted ORs and 95% CIs of MetS for LBP are shown in Table 3. Crude OR (95% CI) of MetS for LBP was 1.20 (0.98–1.45) in men, and 2.27 (1.37–3.63) in women. Even after adjusting for age and lifestyle factors, the associations remained. The interaction of sex and MetS on LBP was statistically significant (*p* = 0.021).

**Table 3:** Association of metabolic syndrome with low back pain

	Total	LBP	Crude		Adjusted*	
	*N*	*n* (%)	OR	95% CI	OR	95% CI
Men						
Mets (−)	25,293	511 (2.0)	1.00		1.00	
Mets (+)	5402	130 (2.4)	1.20	0.98–1.45	1.15	0.95–1.40
Women						
Mets (−)	13,991	228 (1.6)	1.00		1.00	
Mets (+)	506	18 (3.6)	2.27	1.37–3.63	2.16	1.32–3.53

*OR* odds ratio, *CI* confidence interval, *MetS* metabolic syndrome, *LBP* low back pain

*Adjusted for age, smoking habits, alcohol intake, and physical activity

Associations between the clustering of MetS components and LBP are described in Table 4. After adjusting for age and lifestyle factors, men with abdominal obesity had a significantly increased OR for LBP compared to those without abdominal obesity (abdominal obesity alone: OR 1.34, 95% CI 1.02–1.76; abdominal obesity plus one component: OR 1.24, 95% CI 1.01–1.52; abdominal obesity plus two or more components: OR 1.26, 95% CI 1.02–1.55). In contrast, women with abdominal obesity alone did not have significantly increased OR (OR 1.70, 95% CI 0.94–3.08). Adding other components of MetS to abdominal obesity significantly increased the OR for LBP (abdominal obesity plus one component: OR 1.66, 95% CI 1.06–2.60; abdominal obesity plus two or more components: OR 2.30, 95% CI 1.41–3.78).

**Table 4:** Association between the clustering of components of metabolic syndrome and low back pain

	Total	LBP	Crude		Adjusted*	
	*N*	*n* (%)	OR	95% CI	OR	95% CI
Men						
Abdominal obesity (−)	16,651	306 (1.8)	1.00		1.00	
Abdominal obesity (+) + 0	2661	66 (2.5)	1.36	1.04–1.78	1.34	1.02–1.76
Abdominal obesity (+) + 1	5981	139 (2.3)	1.27	1.04–1.56	1.24	1.01–1.52
Abdominal obesity (+) + 2 or more	5402	130 (2.4)	1.32	1.07–1.62	1.26	1.02–1.55
Women						
Abdominal obesity (−)	12,696	194 (1.5)	1.00		1.00	
Abdominal obesity (+) + 0	480	12 (2.5)	1.65	0.92–2.98	1.70	0.94–3.08
Abdominal obesity (+) + 1	815	22 (2.7)	1.79	1.14–2.79	1.66	1.06–2.60
Abdominal obesity (+) + 2 or more	506	18 (3.6)	2.38	1.45–3.89	2.30	1.41–3.78

*OR* odds ratio, *CI* confidence interval, *LBP* low back pain

*Adjusted for age, smoking habits, alcohol intake, and physical activity

## Discussion

The present study investigated the associations of MetS and the clustering of MetS components with LBP among a middle-aged Japanese population. As a result, sex differences in associations were observed; the presence of abdominal obesity was significantly associated with LBP among men, whereas the accumulation of one or more MetS components with abdominal obesity was associated with LBP among women. To the best of our knowledge, this represents the first study to examine sex differences in associations between the clustering of MetS components and LBP.

Our results indicated that the association of MetS with LBP was found in women, but not in men. Ono et al. investigated the association between MetS and LBP among 2650 adults aged 40–74 years in Fukushima Prefecture, Japan [25]. They identified a significant relationship between MetS and LBP in women, but not in men, consistent with our results. A possible explanation for sex differences in the association between MetS and LBP may include disparities in the potency of vascular risk factors. MetS is more likely to influence increased risks for stroke [31, 32] or coronary heart disease [3, 4] in women compared to men. Moreover, several studies have indicated that the effect of MetS on carotid atherosclerosis assessed using ultrasonography was more pronounced in women than in men [1, 2]; this implies that the impact of MetS on atherosclerosis is stronger in women than in men. As one of the underlying mechanisms of LBP, the involvement of atherosclerosis that decreases blood supply to the lumbar region and leads to disc degeneration or LBP has been suggested in several studies (atherosclerosis-LBP hypothesis) [12, 13]. Sex-based differences in the effects of MetS on atherosclerosis might be involved in the results of the present study, although it is difficult to discuss in detail because of lack of imaging tests in lumbar region.

Several recent studies have investigated the effects of the clustering of MetS components on musculoskeletal disorders [6, 8], as well as on CVD or mortality [3, 4]. In addition, the associations of LBP with individual factors of MetS have been investigated, including abdominal obesity [22], hypertension [23], dyslipidemia [17], and impaired blood glucose [24]. However, none have investigated the effect of the accumulation of MetS components on LBP. We therefore analyzed the relationship between clustering of MetS components and LBP. As a result, abdominal obesity increased the OR for LBP among men, irrespective of the further addition of other components. This result may imply that abdominal obesity was an independent factor associated with LBP. Obesity is an important risk factor for LBP; in particular, abdominal obesity might have a great influence on LBP with regard to greater torque and compression loads on the lumbar spine [33]. Moreover, Fan et al. reported that abdominal obesity was significantly associated with atherosclerosis (carotid intima-media thickness) for men only [34]. These studies suggest that our findings were reasonable.

Among women, the addition of one or more MetS components to abdominal obesity significantly increased the OR for LBP, whereas abdominal obesity alone did not show a significantly increased OR for LBP. This result suggests that people who have abdominal obesity and one of the other MetS components, as the “risk group for MetS (pre-MetS) [35],” tend to have LBP. This finding may imply that an intervention for individuals before developing MetS leads to the prevention of LBP. The sex-specific effect of inflammation on MetS might help to interpret the present results. For instance, inflammation has been suggested to be more strongly associated with MetS in women than in men [36, 37]. Moreover, C-reactive protein level, as a marker of inflammation, has been indicated to be higher with an increasing number of MetS components in women than in men [38] and to be a significant predictor of the development of MetS in women [39]. In addition, pro-inflammatory cytokines have been indicated to increase in subjects with LBP and have been suggested to be involved in the pathophysiology of LBP [40, 41]. In these points, further research including biochemical assessment is required to elucidate the relationship.

In the present study, we set LBP under treatment as the outcome of interest. We think that it is important to investigate the characteristics of the disease under treatment from the perspective of public cost. Also in the Comprehensive Survey of Living Conditions, a nationally representative survey conducted by Japanese government (Ministry of Health, Labour and Welfare), many diseases/symptoms under treatment (regardless of treatment options) have been investigated in every 3 years [27]. Moreover, LBP has been indicated to be one of the highest symptoms in the treatment cost (including medical and pharmacy) among health conditions [11]. A better definition of LBP including treatment option which was not provided in this study might have been useful to delineate the relationship between LBP and MetS in more detail.

A key strength in the present study was the large-scale sample (over 40,000 participants), which contributed to decrease the random error. Moreover, although many studies regarding MetS have used body mass index as an alternative to waist circumference, or non-fasting blood to ensure the number of subjects in research [6, 8, 42], we used waist circumference and fasting blood to define MetS accurately. In contrast, some limitations should be noted in this study. First, our study might have the possibility of sampling bias because of a large number of excluded subjects. Differences in characteristics between excluded and included subjects were observed as follows: for example, the proportion of men was 65.8% vs. 67.9% (*p* < 0.001), the proportion of alcohol consumption (everyday) was 33.2% vs. 32.8% (*p* < 0.001), and the proportion of physical activity (≥ 60 min/day) was 33.6% vs. 32.9% (*p* < 0.001). Although these data were statistically significant, the main reason to the observed significance seems to be due to large populations. Second, we used a self-administered questionnaire to collect data on LBP, and no information on specific clinical examinations or disease-specific questionnaires was obtained in this study. Although these assessments may help in delineating the relationship between MetS and LBP, conducting such tests may be difficult in large population-based studies. Third, we could not evaluate several important variables, including occupation type, physical load at work, physical activity earlier in life, menopausal status, or unhealthy lifestyles such as sedentary behavior or sleep disturbance [21, 43–45]. We therefore cannot exclude the possibility of confounding from unmeasured variables in this study. Finally, the causal relationship between MetS and LBP could not be examined due to the cross-sectional nature of the study design. We thus cannot deny the possibility that LBP could contribute to an unhealthy lifestyle, leading to the development of MetS. Prospective studies are needed to examine the causality.

## Conclusions

This large-scale cross-sectional study identified a significant relationship between MetS and LBP in women, but not in men. Moreover, the presence of abdominal obesity was significantly associated with LBP among men, while the accumulation of one or more MetS components along with abdominal obesity was associated with LBP among women. Although further prospective studies are needed to clarify the type of causality, consideration of the clustering of MetS components by sex may be necessary for the prevention of LBP.

## Data Availability

The data used in the current study are available on reasonable request and only after approval by the Ethics Committee of the All Japan Labor Welfare Foundation.

## References

[1] Iglseder B, Cip P, Malaimare L, Ladurner G, Paulweber B (2005). The metabolic syndrome is a stronger risk factor for early carotid atherosclerosis in women than in men. Stroke..

[2] Kawamoto R, Tomita H, Inoue A, Ohtsuka N, Kamitani A (2007). Metabolic syndrome may be a risk factor for early carotid atherosclerosis in women but not in men. J Atheroscler Thromb..

[3] McNeill AM, Rosamond WD, Girman CJ, Golden SH, Schmidt MI, East HE (2005). The metabolic syndrome and 11-year risk of incident cardiovascular disease in the atherosclerosis risk in communities study. Diabetes Care..

[4] Wilson PW, Kannel WB, Silbershatz H, D’Agostino RB (1999). Clustering of metabolic factors and coronary heart disease. Arch Intern Med..

[5] Malik S, Wong ND, Franklin SS, Kamath TV, L’Italien GJ, Pio JR (2004). Impact of the metabolic syndrome on mortality from coronary heart disease, cardiovascular disease, and all causes in United States adults. Circulation..

[6] Yoshimura N, Muraki S, Oka H, Tanaka S, Kawaguchi H, Nakamura K (2012). Accumulation of metabolic risk factors such as overweight, hypertension, dyslipidaemia, and impaired glucose tolerance raises the risk of occurrence and progression of knee osteoarthritis: a 3-year follow-up of the ROAD study. Osteoarthritis Cartilage..

[7] Yoshimura N, Muraki S, Oka H, Tanaka S, Kawaguchi H, Nakamura K (2015). Mutual associations among musculoskeletal diseases and metabolic syndrome components: a 3-year follow-up of the ROAD study. Mod Rheumatol..

[8] Teraguchi M, Yoshimura N, Hashizume H, Muraki S, Yamada H, Oka H (2016). Metabolic syndrome components are associated with intervertebral disc degeneration: the Wakayama Spine Study. PLoS One..

[9] GBD 2017 Disease and Injury Incidence and Prevalence Collaborators (2018). Global, regional, and national incidence, prevalence, and years lived with disability for 354 diseases and injuries for 195 countries and territories, 1990-2017: a systematic analysis for the Global Burden of Disease Study 2017. Lancet.

[10] Dagenais S, Caro J, Haldeman S (2008). A systematic review of low back pain cost of illness studies in the United States and internationally. Spine J..

[11] Loeppke R, Taitel M, Richling D, Parry T, Kessler RC, Hymel P (2007). Health and productivity as a business strategy. J Occup Environ Med..

[12] Kauppila LI (1995). Can low-back pain be due to lumbar-artery disease?. Lancet..

[13] Kauppila LI (2009). Atherosclerosis and disc degeneration/low-back pain--a systematic review. Eur J Vasc Endovasc Surg..

[14] Kurunlahti M, Karppinen J, Haapea M, Niinimaki J, Autio R, Vanharanta H (2004). Three-year follow-up of lumbar artery occlusion with magnetic resonance angiography in patients with sciatica: associations between occlusion and patient-reported symptoms. Spine (Phila Pa 1976)..

[15] Kauppila LI, Tallroth K (1993). Postmortem angiographic findings for arteries supplying the lumbar spine: their relationship to low-back symptoms. J Spinal Disord..

[16] Hemingway H, Shipley M, Stansfeld S, Shannon H, Frank J, Brunner E (1999). Are risk factors for atherothrombotic disease associated with back pain sickness absence? The Whitehall II Study. J Epidemiol Community Health..

[17] Leino-Arjas P, Kaila-Kangas L, Solovieva S, Riihimaki H, Kirjonen J, Reunanen A (2006). Serum lipids and low back pain: an association? A follow-up study of a working population sample. Spine (Phila Pa 1976)..

[18] Heuch I, Heuch I, Hagen K, Zwart JA (2010). Associations between serum lipid levels and chronic low back pain. Epidemiology..

[19] Yoshimoto T, Ochiai H, Shirasawa T, Nagahama S, Kobayashi M, Minoura A (2018). Association between serum lipids and low back pain among a middle-aged Japanese population: a large-scale cross-sectional study. Lipids Health Dis..

[20] Krismer M, van Tulder M (2007). Strategies for prevention and management of musculoskeletal conditions. Low back pain (non-specific). Best Pract Res Clin Rheumatol..

[21] Park YW, Zhu S, Palaniappan L, Heshka S, Carnethon MR, Heymsfield SB (2003). The metabolic syndrome: prevalence and associated risk factor findings in the US population from the Third National Health and Nutrition Examination Survey, 1988-1994. Arch Intern Med..

[22] Briggs MS, Givens DL, Schmitt LC, Taylor CA (2013). Relations of C-reactive protein and obesity to the prevalence and the odds of reporting low back pain. Arch Phys Med Rehabil..

[23] Bae YH, Shin JS, Lee J, Kim MR, Park KB, Cho JH (2015). Association between hypertension and the prevalence of low back pain and osteoarthritis in Koreans: a cross-sectional study. PLoS One..

[24] Real A, Ukogu C, Krishnamoorthy D, Zubizarreta N, Cho SK, Hecht AC (2019). Elevated glycohemoglobin HbA1c is associated with low back pain in nonoverweight diabetics. Spine J..

[25] Ono R, Yamazaki S, Takegami M, Otani K, Sekiguchi M, Onishi Y (2012). Gender difference in association between low back pain and metabolic syndrome: locomotive syndrome and health outcome in Aizu cohort study (LOHAS). Spine (Phila Pa 1976)..

[26] Ministry of Health, Labour and Welfare. Standard questionnaire of specific health checkups. 2018. Available at: http://www.mhlw.go.jp/file/06-Seisakujouhou-10900000-Kenkoukyoku/13_44.pdf. [Accessed 20 Feb 2019].

[27] Myojin T, Ojima T, Kikuchi K, Okada E, Shibata Y, Nakamura M (2017). Orthopedic, ophthalmic, and psychiatric diseases primarily affect activity limitation for Japanese males and females: based on the Comprehensive Survey of Living Conditions. J Epidemiol..

[28] Evaluation Committee on Diagnostic Criteria for Metabolic Syndrome (2005). Metabolic syndrome-definition and diagnostic criteria in Japan. J Jpn Soc Int Med..

[29] Shiri R, Falah-Hassani K (2017). Does leisure time physical activity protect against low back pain? Systematic review and meta-analysis of 36 prospective cohort studies. Br J Sports Med..

[30] Bjorck-van Dijken C, Fjellman-Wiklund A, Hildingsson C (2008). Low back pain, lifestyle factors and physical activity: a population based-study. J Rehabil Med..

[31] Boden-Albala B, Sacco RL, Lee HS, Grahame-Clarke C, Rundek T, Elkind MV (2008). Metabolic syndrome and ischemic stroke risk: Northern Manhattan Study. Stroke..

[32] Takahashi K, Bokura H, Kobayashi S, Iijima K, Nagai A, Yamaguchi S (2007). Metabolic syndrome increases the risk of ischemic stroke in women. Intern Med..

[33] Pryce R, Kriellaars D (2014). Body segment inertial parameters and low back load in individuals with central adiposity. J Biomech..

[34] Fan AZ (2006). Metabolic syndrome and progression of atherosclerosis among middle-aged US adults. J Atheroscler Thromb..

[35] Hamada R, Muto S, Otsuka N, Sato E, Zhang Y (2011). Prevalence of preexisting metabolic syndrome as defined by Japanese original criteria among patients with non-fatal myocardial infarction. Intern Med..

[36] Lai MM, Li CI, Kardia SL, Liu CS, Lin WY, Lee YD (2010). Sex difference in the association of metabolic syndrome with high sensitivity C-reactive protein in a Taiwanese population. BMC Public Health..

[37] Ren Z, Zhao A, Wang Y, Meng L, Szeto IM, Li T, et al. Association between dietary inflammatory index, C-reactive protein and metabolic syndrome: a cross-sectional study. Nutrients. 2018;10. 10.3390/nu10070831 PMC6073906 29954070

[38] Rutter MK, Meigs JB, Sullivan LM, D'Agostino RB, Wilson PW (2004). C-reactive protein, the metabolic syndrome, and prediction of cardiovascular events in the Framingham Offspring Study. Circulation..

[39] Han TS, Sattar N, Williams K, Gonzalez-Villalpando C, Lean ME, Haffner SM (2002). Prospective study of C-reactive protein in relation to the development of diabetes and metabolic syndrome in the Mexico City Diabetes Study. Diabetes Care..

[40] Wang H, Schiltenwolf M, Buchner M (2008). The role of TNF-alpha in patients with chronic low back pain-a prospective comparative longitudinal study. Clin J Pain..

[41] Li Y., Liu J., Liu Z.-z., Duan D.-p. (2016). Inflammation in low back pain may be detected from the peripheral blood: suggestions for biomarker. Bioscience Reports.

[42] Irie F, Iso H, Noda H, Sairenchi T, Otaka E, Yamagishi K (2009). Associations between metabolic syndrome and mortality from cardiovascular disease in Japanese general population, findings on overweight and non-overweight individuals. Ibaraki Prefectural Health Study. Circ J..

[43] Barone Gibbs B, Hergenroeder AL, Perdomo SJ, Kowalsky RJ, Delitto A, Jakicic JM (2018). Reducing sedentary behaviour to decrease chronic low back pain: the stand back randomised trial. Occup Environ Med..

[44] Kelly GA, Blake C, Power CK, O'Keeffe D, Fullen BM (2011). The association between chronic low back pain and sleep: a systematic review. Clin J Pain..

[45] Coenen P, Gouttebarge V, van der Burght AS, van Dieën JH, Frings-Dresen MH, van der Beek AJ (2014). The effect of lifting during work on low back pain: a health impact assessment based on a meta-analysis. Occup Environ Med..

